# High-Frequency, Low-Magnitude Vibration Does Not Prevent Bone Loss Resulting from Muscle Disuse in Mice following Botulinum Toxin Injection

**DOI:** 10.1371/journal.pone.0036486

**Published:** 2012-05-10

**Authors:** Sarah L. Manske, Craig A. Good, Ronald F. Zernicke, Steven K. Boyd

**Affiliations:** 1 Faculty of Kinesiology, University of Calgary, Calgary, Canada; 2 Schulich School of Engineering, University of Calgary, Calgary, Canada; 3 Faculty of Medicine, University of Calgary, Calgary, Canada; 4 Departments of Orthopaedic Surgery and Biomedical Engineering and School of Kinesiology, University of Michigan, Ann Arbor, Michigan, United States of America; University of Zaragoza, Spain

## Abstract

High-frequency, low-magnitude vibration enhances bone formation ostensibly by mimicking normal postural muscle activity. We tested this hypothesis by examining whether daily exposure to low-magnitude vibration (VIB) would maintain bone in a muscle disuse model with botulinum toxin type A (BTX). Female 16–18 wk old BALB/c mice (N = 36) were assigned to BTX-VIB, BTX-SHAM, VIB, or SHAM. BTX mice were injected with BTX (20 µL; 1 U/100 g body mass) into the left hindlimb posterior musculature. All mice were anaesthetized for 20 min/d, 5 d/wk, for 3 wk, and the left leg mounted to a holder. Through the holder, VIB mice received 45 Hz, ±0.6 g sinusoidal acceleration without weight bearing. SHAM mice received no vibration. At baseline and 3 wk, muscle cross-sectional area (MCSA) and tibial bone properties (epiphysis, metaphysis and diaphysis) were assessed by *in vivo* micro-CT. Bone volume fraction in the metaphysis decreased 12±9% and 7±6% in BTX-VIB and BTX-SHAM, but increased in the VIB and SHAM. There were no differences in dynamic histomorphometry outcomes between BTX-VIB and BTX nor between VIB and SHAM. Thus, vibration did not prevent bone loss induced by a rapid decline in muscle activity nor produce an anabolic effect in normal mice. The daily loading duration was shorter than would be expected from postural muscle activity, and may have been insufficient to prevent bone loss. Based on the approach used in this study, vibration does not prevent bone loss in the absence of muscle activity induced by BTX.

## Introduction

High strain magnitudes (>1000 με) and strain rates are well documented characteristics of an osteogenic mechanical stimulus [1], [2]. However, evidence suggests that lower magnitude vibration (<1 g acceleration) applied at higher frequencies (>10 Hz) also promotes osteogenesis [3], [4]. The application of low-magnitude vibratory stimuli to prevent bone loss was initiated, in part, due to the high prevalence of low strain magnitude events observed in bone during daily activities [5]. Further, there is an inverse, non-linear relation between the incidence of events and magnitude of strain that maintain bone mass, suggesting that loading regimes performed at a sufficiently large number of cycles per day, at lower strain magnitudes may elicit an osteogenic response [6]. These findings suggested that low-magnitude, rather than high-magnitude, strain events can drive osteogenesis and perhaps prevent bone loss.

Many studies have since demonstrated that low-magnitude vibration is osteogenic in animals [3], [7], [8], [9], [10], [11] and in humans who comply with the vibration training protocol (1–2 sessions x 10 minutes/day) [12], [13]. However, other studies applying similar protocols in animals and humans failed to demonstrate a positive effect of low-magnitude vibration [14], [15], [16]. These conflicting results indicate that the mechanisms and parameters (i.e., frequency, strain and, acceleration amplitude) by which high-frequency, low-magnitude vibration drives bone formation are poorly understood. A better understanding of the mechanism underlying bone's ability to adapt to low-magnitude mechanical signals is needed to design effective interventions to prevent or mitigate bone loss.

One potential source of endogenous high-frequency, low-magnitude strains observed in bone may be muscle activity [3], [17]. Muscles are known to play an important role in transmitting mechanical stimuli to bone [18], [19], and postural muscles emit high-frequency vibrations (10–50 Hz) when activated [20]. Further, the frequency content in the 30–50 Hz range, associated with the firing rates of fast-twitch muscle fibres, decline with age [20]. This decline, related to the preferential decrease of fast-twitch muscle fibres with age, may contribute to age-related bone loss. Based on these collective findings, Rubin and colleagues [5], [6], [20] postulated that bone mass may be maintained as a result of high-frequency, low-magnitude strains produced by postural muscle activity, and that exogenous high-frequency, low-magnitude vibration may promote bone adaptation by mimicking postural muscle activity. However, to our knowledge, the proposition that bone is maintained by postural muscle activity has not been examined.

While most studies have delivered vibration to the lower limbs via whole body vibration (WBV), recent findings suggest that weight-bearing and muscle activity may not be required to induce an osteogenic response to low-magnitude vibration [21], [22], [23]. Specifically, bone formation rates increased when vibration was delivered to a single hindlimb while a mouse was anaesthetized, regardless of whether the mouse was exposed to normal ambulation [22] or disuse as a result of hindlimb unloading [21] for the rest of the day.

Another means of trigerring disuse is by inhibiting muscle activation with injection of botulinum toxin type A (BTX). We, and others, have previously demonstrated that BTX-induced muscle and bone loss occurs within one week post-injection [24], and is maintained for at least four weeks post-injection [25], [26]. Further, BTX-induced bone loss appears to occur primarily as a result of muscle atrophy [27] and has minimal effects on gait [28] and weight-bearing ability in the injected limb [29].

Therefore, we hypothesized that daily exposure to high-frequency, low-magnitude vibration would maintain bone structure despite muscle disuse in mice. Further, we wanted to confirm the result that vibration could increase bone formation in healthy, normal mice. To test this hypothesis, we triggered muscle disuse in skeletally mature mice by injection with BTX. We applied daily exposure to vibration without weight-bearing, using an osteogenic protocol designed by Garman et al. [21], [22].

## Materials and Methods

### Animal Model and Experimental Design

**Figure 1:**
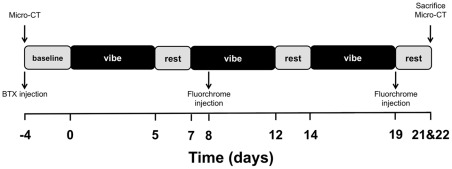
Diagram of the experimental protocol.

This experiment was conducted in two parts. In both parts female, 16–18 week old, skeletally mature BALB/cAnNCrl (BALB) mice [30], [31] were obtained from Charles River Laboratories (Saint-Constant, Quebec, Canada). For Experiment 1, 27 mice were assigned to one of the following groups: high-frequency, low-magnitude vibration + injection with botulinum toxin type A (BTX-VIB, n = 9), BTX-SHAM (n = 8), or SHAM (n = 8). For Experiment 2, 11 mice were assigned to high-frequency, low-magnitude vibration (VIB, n = 8) or SHAM (n = 3). Group assignment was performed to ensure consistent mean body mass between the groups. The animals for each experiment were obtained at different dates, and thus there were small differences in baseline body mass between experiments. Additional mice (n = 2) were sacrificed prior to commencing Experiment 1 for strain measurements. BTX mice were injected with 20 μL of BTX Type A (BOTOX, Allergan Inc., 1 U/100 g) into the left posterior lower limb musculature (single injection targeting gastrocnemius, plantaris, and soleus) immediately following baseline measurements. Beginning 4 days after baseline measurements, VIB mice received a high-frequency (45 Hz), low-magnitude mechanical stimulus to the left, treated, tibia. The stimulus was sinusoidal and delivered with a peak acceleration of ±0.6 g. The right, untreated, leg was not loaded and remained on the bed. SHAM mice were handled and attached to the oscillatory device, but the vibration stimulus was not applied. The vibration and sham procedures were conducted with the mice under isoflurane anaesthesia for 20 minutes/day, 5 days/week for 3 weeks. The timeline of the experimental protocol is outlined in Figure 1.

All animals were housed in groups and provided standard rodent chow and water *ad libitum*. Following BTX injection, BTX mice were provided with food and Napa Nectar (SE Lab Group, Napa, CA) on the cage bottom to ease access to food for one week post-injection. Otherwise, normal cage activity with normal weight-bearing was permitted. Body mass was monitored after each vibration session and reported weekly. Micro-computed tomography (micro-CT) scans were performed at baseline and prior to sacrifice on day 21. To assess dynamic indices of bone formation, in Experiment 1, mice were injected with calcein green (1 mg/100 g; IP) on days 8 and 19. In Experiment 2, mice were injected with calcein green on day 8 and oxytetracycline (2.5 mg/kg; IP) on day 19. Mice were sacrificed over two days (days 21 and 22), to allow time for scanning and tissue processing, via cervical dislocation while under anaesthesia. All procedures were approved by the Health Sciences Animal Care Committee at the University of Calgary.

### Application of Low-Magnitude Mechanical Stimulus

**Figure 2:**
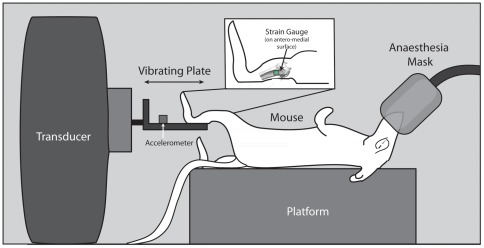
Apparatus used to apply high-frequency, low-magnitude vibration to the lower limb of mouse, along the longitudinal axis of the tibia. A strap (not shown) held the hindlimb in place.

Application of the high-frequency, low-magnitude mechanical stimulus was performed in the absence of weight-bearing. The protocol was designed to replicate that described by Garman et al. [21], [22], as they detected positive effects of vibration without weight-bearing in the proximal tibia epiphysis with micro-CT. The apparatus to deliver the displacements included a vibration shaker (V203, Ling Dynamic Systems, Royston, England) driven by an amplified sinusoidal tone generator signal (Power Amplifier Type 2706, Brüel & Kjær, Nærum, Denmark; NCH Tone Generator v2.12, Canberra, Australia). The mouse was positioned in a bed on its back, and the distal portion of the left leg fixed to a holder with a strap, with the tibio-femoral angle at approximately 120°. The vibration was applied along the longitudinal axis of the tibia (Figure 2).

Acceleration was measured throughout the vibration protocol using a uniaxial accelerometer (EGAX-10, Entran Devices, range ±10 g, Fairfield, NJ, USA) fixed to the holder between the leg and shaker. Acceleration signals were amplified (2310A Signal Conditioning Amplifier, Measurements Group Inc.), sampled at 1000 Hz (DATAQ Instruments Model DI-205, Akron, OH), and displayed on a computer screen (WinDaq Acquisition DI 720, DATAQ Instruments, Akron, OH) for continuous monitoring.

### Strain Quantification

Strain measurements were performed in two mice immediately after sacrifice, prior to commencing the study. The anterior surface of the proximal tibia diaphysis was minimally cleaned of soft tissue, and a single element strain gauge (FLK-1–11, Tokyo Sokki Kenkyujo Co., Tokyo, Japan) was fixed to the anterior tibia using cyanoacrylate strain gauge adhesive (CN adhesive, Tokyo Sokki Kenkyujo Co., Tokyo, Japan). The longitudinal axis of the strain gauge was aligned with the longitudinal axis of the tibia. Strains were amplified (2310A Signal Conditioning Amplifier, Measurements Group Inc., Raleigh, NC, USA), and sampled at 1000 or 2000 Hz (WinDaq Acquisition DI 720, DATAQ Instruments Model DI-205, DATAQ Instruments, Akron, OH, USA) during 3 trials with 45 Hz, ±0.6 g sinusoid, and with no signal applied. Using MATLAB (R2008a, Natick, MA, USA), strain signals were filtered with a 200 Hz 2^nd^ order Butterworth low-pass filter (using the function ‘filtfilt’) [5], [21]. Peak-to-peak strains and frequency content of the resulting signal with the Fast Fourier Transform function (FFT).

### *In vivo* Micro-CT

An *in vivo* micro-CT scanner (vivaCT 40, Scanco Medical, Brüttisellen, Switzerland) quantified muscle cross-sectional area (MCSA) and bone outcomes. Mice were anaesthetized with isoflurane and maintained on anaesthetic gases for the duration of the scan. The hindlimbs were positioned and scanned in parallel with a custom fixture to obtain images of both limbs [27]. Scans were performed at the proximal tibia epiphysis and metaphysis to replicate the regions examined by Garman et al. [21], as well as the proximal tibia diaphysis. The procedure described briefly below follows methods used previously for scans obtained at baseline and 3 weeks [27], [29].

### Muscle Outcomes

#### *In-vivo* Muscle Measurements

MCSA (mm^2^) was measured in the diaphyseal region of each lower hindlimb using *in vivo* micro-CT. The volume scanned was a 2.65 mm slab encompassing the maximal MCSA, as determined in pilot experiments, beginning 2.2 mm distal to the tibial growth plate. The scanning parameters used were 45 kVP, 133 μA, 620 ms integration time, 250 projections/180° resulting in a 50 μm isotropic voxel size and a total scan time of 6 minutes [32].

#### Image Processing

For each hindlimb a 0.8 mm slab was extracted from the gray-scale images and Gaussian filtered (σ = 1.2, support  = 2). All micro-CT image intensities are expressed as a fixed fraction of the maximal gray-scale value (1000). Three threshold values were used to produce segmented images of the entire leg (2.4% of maximal gray value), muscle region excluding the subcutaneous fat (10.6% of maximal gray value), and bone (15% of maximal gray value).

#### *Ex-vivo* Muscle Measurements

Upon sacrifice, the posterior compartment muscles (gastrocnemius and soleus) and tibialis anterior were dissected, and wet muscle mass (mg) was measured.

### Bone Outcomes

Each proximal tibial micro-CT measurement examined a 5.3 mm thick slab, corresponding to 424 slices encompassing the proximal tibia epiphysis and metaphysis. The scanning parameters used were 55 kVP, 133 μA, 200 ms integration time, 2048 samples and 1000 projections/180°, resulting in a 12.5 μm isotropic voxel size and a total scan time of 20 minutes. The proximal tibia diaphysis measurement was obtained during the muscle scan.

#### Image Processing

Volumes extracted from each limb included the proximal tibia epiphysis (encompassing the entire epiphysis), the proximal tibia metaphysis (a 1 mm slab extending distally from the growth plate), and the proximal tibia diaphysis (a 0.8 mm slab beginning 2.2 mm distal to the tibial growth plate). Hand-drawn contours were used to isolate the metaphyseal region of interest; trabecular and cortical compartments were segmented using an automated method [33]. After segmentation, the resulting gray-scale images were Gaussian filtered (σ = 1.2, support  = 2). A global threshold was applied (27.5% of maximal gray-scale value for diaphyseal volumes, 30% of maximal gray-scale value for metaphyseal and epiphyseal volumes) to form binarized images on which morphological analyses were performed.

#### Morphological Analysis of Micro-CT Scans

Bone micro-architecture was assessed with direct 3D methods (Image Processing Language v. 5.07b; Scanco) [34]. Morphological measurements in the trabecular region of the epiphysis and metaphysis included total bone volume (BV, mm^3^), trabecular bone volume fraction (BV/TV, %), trabecular thickness (Tb.Th, mm), trabecular separation (Tb.Sp, mm), and trabecular number (Tb.N, mm^−1^). In addition, several non-metric parameters were assessed including structural model index (SMI) [35], connectivity density (ConnD, mm^−3^) [36], and degree of anisotropy (DA). In the cortical region of the metaphysis and diaphysis, cortical area (Ct.Ar, mm^2^) was calculated, excluding porosity, as well as total area within the periosteal envelope (Tt.Ar, mm^2^), and area within the marrow cavity (Ma.Ar, mm^2^).

#### Dynamic Histomorphometry

After sacrifice, the tibiae were dissected, cleared of soft tissue, and fixed in 70% ETOH. Subsequently, the left, treated, tibiae from all mice were dehydrated and embedded, undecalcified, in poly methyl metacrylate (PMMA), thick sectioned in the axial plane using a diamond band saw (EXAKT, Norderstedt, Germany), and ground to 100 μm thickness. Sections were visualized using epifluorescence with near-infrared filters on a Axiovert system (Zeiss, Jena, Germany). Within the trabecular bone region, single-labeled surface (sLS), double-labeled surface (dLS), and bone surface (BS) were measured with Image-Pro AMS (v5.1.2.59, Media Cybernetics, Bethesda, MD, USA). Interlabel thickness (Ir.L.Th, μm) was also measured with Bioquant Osteo (Bioquant Image Analysis Corporation, Nashville TN, USA) or Osteomeasure (Osteometrics, Decatur GA, USA). From these measures, percent single-labeled surface (%sLS), percent double-labeled surface (%dLS), mineralizing surface (MS  =  (dLS + sLS/2)/BS, %), mineral apposition rate (MAR  =  Ir.L.Th/Ir.L.T, μm/d, with Ir.L.T  =  interlabel period), and bone formation rate (BFR  =  MAR * MS/BS, μm^3^/μm^2^/y) were calculated [37].

### Statistical Analysis

Changes in whole body mass were examined with a two-way analysis of variance (ANOVA) with Group (BTX-VIB, BTX-SHAM, VIB, SHAM) as a between-subject factor and Time as a within-subject factor. Mean change from baseline was used as the outcome for all micro-CT variables. To examine changes in micro-CT variables and muscle mass, two-way ANOVAs were performed with Group (BTX-VIB, BTX-SHAM, VIB, SHAM) as a between-subject factor and Leg (treated, untreated) as a within-subject factor. Simple effect testing with a Bonferroni correction was used to interpret significant interactions. Differences in bone histomorphometry were examined with a one-way ANOVA with Group as a between-subject factor and Tukey's post-hoc test. IBM SPSS Statistics version 19.0 (SPSS Inc., Chicago) was used for all statistical analyses. Values are expressed as mean ± SD in tables and mean ± SE in the figures for clarity. The significance level was set at p<0.05. Effect size is reported as the partial eta-squared (η^2^) for the interaction effects as well as simple effects and post-hoc tests to estimate the proportion of variability associated with the comparison of interest [38].

## Results

### Strain Measurement

**Figure 3:**
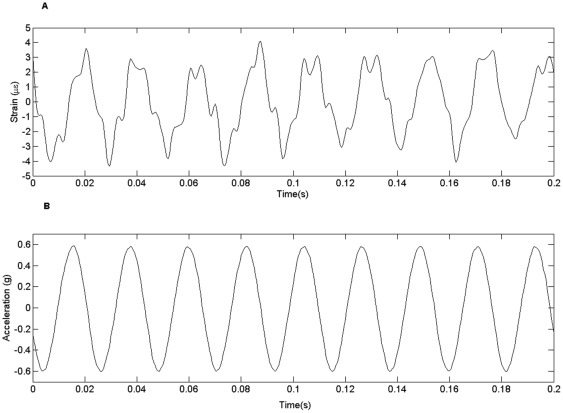
Strain (**A**) **and acceleration** (**B**) **vs. time for a 0.2** **s portion of one trial.** The strain gauge was fixed to the anterior surface of the proximal tibia. The accelerometer was fixed to the leg holder.

A sinusoidal, 45 Hz vibration signal with ±0.6 g acceleration induced peak-to-peak strains of 5.8±0.3 με with a mean of zero on the proximal tibia recorded in several trials on two mice (Figure 3A). There was a slight lag or phase shift between recorded acceleration and strain (Figure 3B). The phase shift is consistent with the soft tissue compliance effects between the vibration shaker and the strain measurement location. The predominant frequency peak in the spectrum of the strain recording with ±0.6 g acceleration input was 45 Hz. In contrast, there was no discernable strain signal when vibration was not applied.

### Body Mass and Muscle Outcomes

Body mass decreased in both BTX groups after baseline (Group x Time interaction, p<0.001, partial η^2^ = 0.63). At day 21, body mass remained 4.4±1.2% lower than baseline in the BTX-VIB group (p<0.001, parital η^2^ = 0.97) and 6.2±2.4% lower than baseline in the BTX-SHAM group (p<0.001, parital η^2^ = 0.96). Body mass did not change significantly in the SHAM but increased 3.7±0.8% (p = 0.003, partial η^2^ = 0.97) in the VIB groups.

There was a significant Group x Leg interaction for MCSA (p<0.001, partial η^2^ = 0.94). Within-group change in MCSA was not significantly different between the BTX-VIB and BTX-SHAM groups (Table 1, p>0.05, partial η^2^ = 0.07). However, MCSA decreased in the treated leg of the BTX-VIB group by 47.6±3.4%, and in the BTX-SHAM group by 46.1±3.5%, and these declines were significantly greater than the SHAM group (p<0.001, partial η^2^ = 0.93 and p<0.001, partial η^2^ = 0.91, respectively). While there was a trend towards a greater increase in MCSA in the treated leg of the VIB than SHAM group (p = 0.16, partial η^2^ = 0.18), and a significantly greater increase in the untreated leg of the VIB than SHAM groups (p = 0.004, partial η^2^ = 0.27), this was likely associated with the slightly lower baseline MCSA in the mice used in Experiment 2. There was also a significant decrease in MCSA in the untreated leg of the BTX-VIB group compared with the SHAM group (p = 0.02, partial η^2^ = 0.47).

**Table 1:** Effect of treatment (BTX-VIB, BTX-SHAM, VIB, SHAM) on muscle cross-sectional area (mm^2^).

Leg	Treatment	Baseline	Day 21
Treated	BTX-VIB	72.8±3.7	38.2±3.9^a,b^
	BTX-SHAM	72.7±2.4	39.2±3.9^a,b^
	VIB	68.3±4.5	73.2±2.6
	SHAM	73.0±5.3	73.5±4.2
Untreated	BTX-VIB	72.0±4.5	67.8±4.0^c,d^
	BTX-SHAM	71.7±2.2	68.6±4.1^c,d^
	VIB	68.3±2.8	72.7±3.9
	SHAM	71.9±5.5	74.5±3.0^c^

Significant between-group differences (p<0.05) for change between baseline and 21 d, determined by simple effect tests with Bonferroni adjustments are indicated by the following: ^a^Group change is significantly different than treated VIB leg; ^b^Group change is significantly different than treated SHAM leg; ^c^Group change is significantly different than untreated VIB leg; ^d^ Group change is significantly different than untreated SHAM leg. Values are the unadjusted mean ± SD (n = 8 for BTX-SHAM and VIB; n = 9 for BTX-VIB; n = 11 for SHAM).

Similarly, gastrocnemius, soleus, and tibialis anterior muscle masses were significantly lower in the treated leg of the BTX-VIB and BTX-SHAM groups compared with the SHAM and VIB groups (significant interactions, p<0.001 for all significant comparisons, Table 2). However, tibialis anterior mass was significantly higher in the BTX-VIB than BTX group (p = 0.04, partial η^2^ = 0.30). Gastrocnemius mass was also significantly lower in the untreated leg of BTX-VIB and BTX-SHAM than the SHAM group (p<0.001 and p = 0.005, respectively, partial η^2^ = 0.97). There were no differences in muscle mass between the VIB and SHAM groups.

**Table 2:** Effect of treatment (BTX-VIB, BTX-SHAM, VIB, SHAM) on individual muscle masses (mg) 21 days following treatment.

		Posterior		Anterior
Leg	Treatment	Gastrocnemius	Soleus	Tibialis Anterior
Treated	BTX-VIB	29.7±4.7^a,b^	3.7±0.6^a,b^	27.4±2.7^a,b,c^
	BTX- SHAM	34.0±9.7^a,b^	3.9±0.7^a,b^	20.8±7.4^a,b^
	VIB	101.3±6.8	6.8±0.8	41.6±1.8
	SHAM	100.3±5.4	6.3±1.1	41.1±5.1
Untreated	BTX- VIB	89.6±5.0^d^	7.0±0.5	36.6±2.4
	BTX- SHAM	92.5±5.8^d^	6.4±0.6	36.3±2.7
	VIB	102.2±4.0	6.7±0.8	38.6±1.7
	SHAM	103.5±7.0	6.5±0.8	38.6±2.5

Significant Group x Leg interaction for muscle mass (p<0.001, partial η^2^ = 0.81 to 0.96). Significantly lower muscle masses (p<0.05) determined by simple effect tests with Bonferroni adjustments are indicated by the following: ^a^ compared with untreated leg; ^b^ compared with SHAM and VIB treated legs (partial η^2^ = 0.93 to 0.99); ^c^ compared with BTX-SHAM treated leg (partial η^2^ = 0.30);^ d^ compared with SHAM and VIB untreated leg (partial η^2^ = 0.95 to 0.97). Values are the mean ± SD (n = 8 for BTX-SHAM and VIB; n = 9 for BTX-VIB; n = 11 for SHAM).

### Bone Outcomes

**Figure 4:**
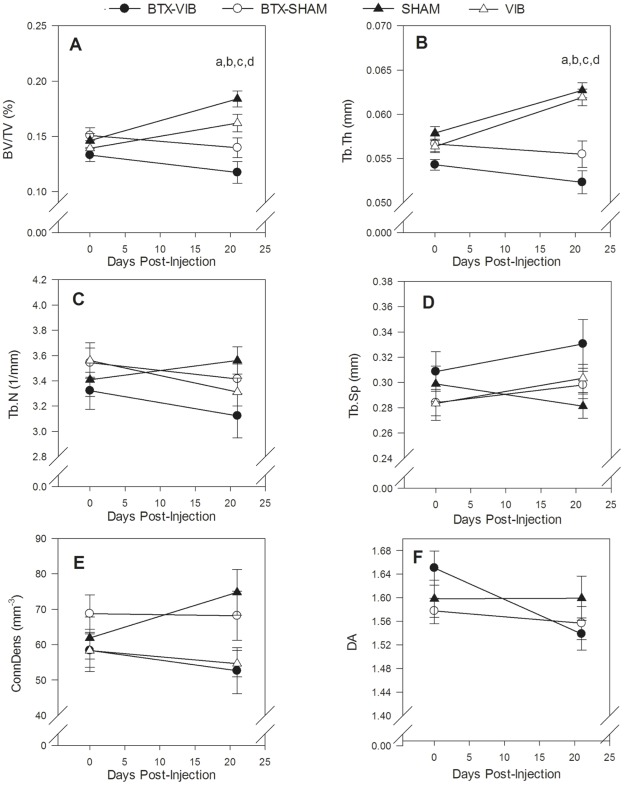
Effect of treatment on bone micro-architecture in the proximal tibial metaphysis on the treated (**left**) **hindlimb.** Significant Group x Leg interactions for change between baseline and 21 d for BV/TV (p<0.001, partial η^2^ = 0.51), and TbTh (p<0.001, partial η^2^ = 0.64). Significant between-group differences (p<0.05) determined by simple effect tests with Bonferroni adjustments are indicated by the following: ^a^ BTX-VIB < SHAM; ^b^ BTX-SHAM < SHAM; ^c^ BTX-VIB < VIB; ^d^ BTX-SHAM < VIB. Values are the unadjusted mean ± SE (n = 8 for BTX-SHAM and SHAM; n = 9 for BTX-VIB, n = 11 for VIB).

**Figure 5:**
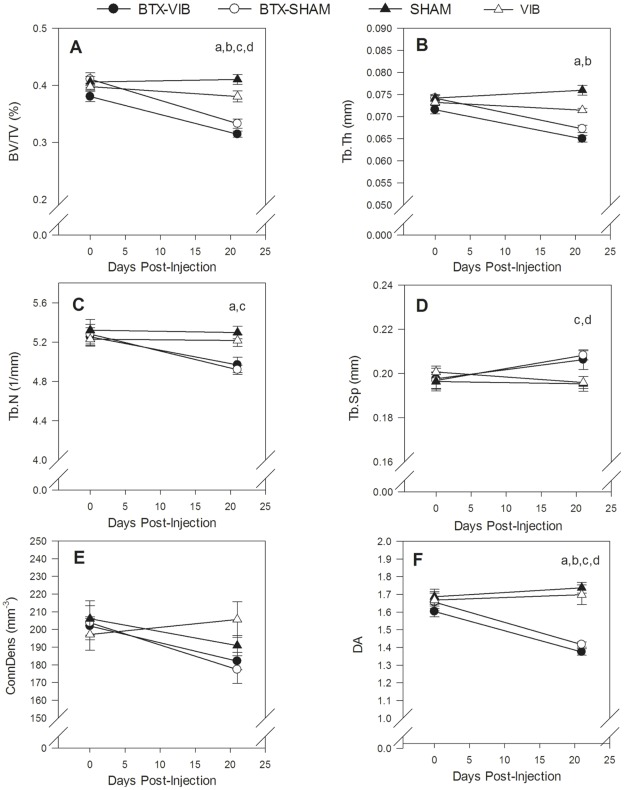
Effect of treatment on bone micro-architecture in the proximal tibial epiphysis of the treated (**left**) **hindlimb.** Significant Group x Leg interactions for change between baseline and 21d for BV/TV (p<0.001, partial η^2^ = 0.42), TbTh (p<0.001, partial η^2^ = 0.34) and DA (p<0.001, partial η^2^ = 0.46). Significant between-group differences (p<0.05) determined by simple effect tests with Bonferroni adjustments are indicated by the following: ^a^ BTX-VIB < SHAM; ^b^ BTX-SHAM < SHAM; ^c^ BTX-VIB < VIB; ^d^ BTX-SHAM < VIB. Values are the unadjusted mean ± SE (n = 8 for BTX-SHAM and SHAM; n = 9 for BTX-VIB, n = 11 for VIB).

Bone micro-architecture declined in the treated leg of both BTX groups. In the proximal tibia metaphysis, BV/TV decreased 11.8±8.5% in the treated leg of the BTX-VIB group and 7.3±6.4% in the BTX-SHAM group, but increased 25.8±3.9% in the treated leg of the SHAM group and 16.4±4.9% in the VIB group. These within-group decreases in the BTX groups were significantly different from the SHAM (p<0.001, partial η^2^ = 0.51) and VIB groups (p = 0.01, partial η^2^ = 0.04 compared with BTX-VIB and p = 0.02, partial η^2^ = 0.02 compared with BTX-SHAM, Figure **4**A). Similarly, the within-group decreases in metaphyseal Tb.Th in the treated leg of both BTX groups differed from the SHAM and VIB groups (p<0.003, partial η^2^ >0.51 for all significant comparisons, Figure **4**B). We observed similar trends between groups in the proximal tibia epiphysis (Figure **5**). The within-group decreases in BV/TV in the treated leg of the BTX-VIB group (17±1.7%) and BTX-SHAM group (19±2.4%) differed significantly from the SHAM (p<0.001, partial η^2^ = 0.33) and VIB groups (p = 0.02, η^2^ = 0.41 compared with BTX-VIB and p = 0.002, partial η^2^ = 0.39 compared with BTX-SHAM, Figure **5**A). Similarly, the within-group decreases in epiphyseal Tb.Th (p<0.001, partial η^2^ = 0.38, Figure **5**B) and DA (p<0.001, partial η^2^ = 0.39, Figure **5**F) in the treated leg of the BTX groups were significantly different from the SHAM group. There were no significant differences in within-group change of any micro-architectural parameters between the treated leg of the BTX-VIB and BTX-SHAM groups, nor VIB and SHAM groups in the tibia metaphysis (partial η^2^<0.01) or epiphysis (partial η^2^<0.02).

**Figure 6:**
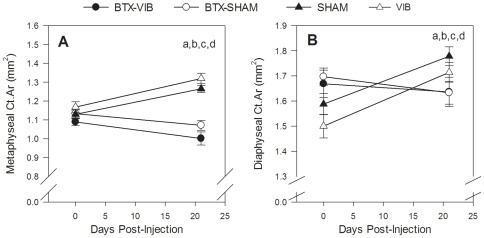
Effect of treatment on Ct.Ar in the proximal tibial metaphysis (A) and diaphysis (B) of the treated (left) hindlimb. Significant Group x Leg interactions for change between baseline and 21 d for metaphysis Ct.Ar (p<0.001, partial η^2^ = 0.72), diaphysis Ct.Ar (p = 0.10, partial η^2^ = 0.30). Significant between-group differences (p<0.05) determined by simple effect tests with Bonferroni adjustments are indicated by the following: ^a^ BTX-VIB < SHAM; ^b^ BTX-SHAM < SHAM; ^c^ BTX-VIB < VIB; ^d^ BTX-SHAM < VIB. Values are the unadjusted mean ± SE (n = 8 for BTX-SHAM and SHAM; n = 9 for BTX-VIB, n = 11 for VIB).

The within-group change in cortical bone architecture was significantly different in the treated leg of the BTX groups than the SHAM group (Figure **6**). Metaphyseal Ct.Ar declined 8.1±3.5% and 5.5±2.5% in the BTX-VIB and BTX-SHAM groups, respectively, but increased 12.0±1.5% in the SHAM group and 12.9±2.1% in the VIB group and these within-group decreases in the BTX groups were significantly different from the SHAM (p<0.001, partial η^2^ = 0.8) and VIB groups (p<0.001, partial η^2^ = 0.09 to 0.18, Figure **6**A). The decline in metaphyseal Ct.Ar occurred primarily as a result of a significant increase in Ma.Ar in the BTX groups (data not shown, p<0.001, partial η^2^ = 0.53 for Group x Leg interaction, p<0.002 for all significant comparisons, partial η^2^ = 0.16 to 0.51). The 1.9±2.6% and 3.7±2.8% decrease in diaphyseal Ct.Ar in the BTX-VIB and BTX-SHAM groups, respectively, was significantly different from the within-group changes in the SHAM and VIB groups (p<0.001 for all significant comparisons, partial η^2^ = 0.39 to 0.43, Figure **6**B). There were no significant differences in change in cortical area between the treated leg of the BTX-VIB and BTX-SHAM groups (partial η^2^ = 0.003 to 0.024), nor VIB and SHAM (partial η^2^ = 0.39 to 0.71) groups.

There were no visible double labels in three samples examined (n = 1 from BTX-SHAM, n = 2 from SHAM), and thus they were excluded from the analysis. Processing errors led to exclusion of four additional samples (n = 1 from each group). Significant ANOVAs (p<0.05, partial η^2^ = 0.36 to 0.40) and Tukey's post-hoc tests indicated that BTX+VIB had significantly lower %dLS (p = 0.04, partial η^2^ = 0.09), MS/BS (p = 0.04, partial η^2^ = 0.27), and BFR/BS (p = 0.04, partial η^2^ = 0.22) than VIB, and significantly lower %dLS (p = 0.01, partial η^2^ = 0.70) and BFR/BS (p = 0.01, partial η^2^ = 0.74) than SHAM (Table 3). There were no differences in any histomorphometric indices between the BTX-VIB and BTX-SHAM groups (partial η^2^ = 0.002 to 0.091), nor VIB and SHAM groups (partial η^2^ = 0.77 to 0.96).

**Table 3:** Effect of treatment on static and dynamic histomorphometric indices in the trabecular region of the proximal tibia metaphysis in the treated leg.

Treatment	sLS/BS	dLS/BS	MS/BS	MAR	BFR/BS
	(%)	(%)	(%)	(μm/d)	(μm^3^/μm^2^/y)
BTX-VIB	19.4±15.2	5.6±4.4^a^	15.3±9.2^a^	1.33±0.25	70.2±35.7a,b
BTX-SHAM	15.4±2.4	7.4±4.8^b^	15.1±4.8	1.32±0.17	73.2±25.0
VIB	28.1±11.2	16.2±9.7	30.3±14.5	1.32±0.28	150.6±83.0
SHAM	20.7±5.9	17.8±8.4	28.2±8.7	1.49±0.21	152.0±50.7

a Group is significantly lower than treated VIB leg (p<0.05); ^b^ Group is significantly lower than treated SHAM leg (p<0.01). Values are the mean ± SD (n = 8 for BTX-VIB and SHAM; n = 6 for BTX; n = 7 for VIB). Four samples were excluded due to processing errors. Samples (n = 3) without double labels were also excluded. sLS/BS  =  % single-labelled surface; dLS/BS  =  % double labelled surface; MS/BS  =  % mineralizing surface; MAR  =  mineral apposition rate; BFR/BS  =  bone formation rate, normalized by bone surface.

## Discussion

In this study, we found that 20 minutes of daily exposure to high-frequency, low-magnitude vibration did not prevent muscle and bone loss associated with BTX injection in BALB mice. Further, we were unable to identify an anabolic effect of vibration in either the presence or absence of muscle activity. Our results do not lend support to the hypothesis that high-frequency, low-magnitude vibration mimics the effects of postural muscle activity on bone.

There are several possible explanations for our finding that vibration did not prevent or attenuate BTX-induced bone loss. Similar to our result, Brouwers et al. [15] found that WBV applied at 90 Hz, 0.3 g, for two 20 minute sessions/day for 6 weeks could not attenuate bone loss in rats after ovariectomy. In contrast, previous studies by Garman et al. found that neither muscle activity nor weight-bearing were required for vibration to stimulate bone formation in animals undergoing normal loading [21] or hindlimb unloading [22] during the other hours of the day. The primary difference between the Garman et al. [22] study and the current study was the use of BTX rather than hindlimb unloading to trigger disuse to specifically test the hypothesis that vibration mimics the effects of muscle activity on bone. However, there were several similarities between the studies. In both studies, voluntary muscle activity was absent during vibration loading because the animals were under anaesthesia. Further, the magnitude of bone loss (or attenuation of bone gain) appeared to be similar following 3 weeks disuse due to hindlimb unloading (∼49% of control value) or BTX (∼61% of control value after BTX). However, there are differences in daily muscle activity in animals subjected to hindlimb unloading compared with BTX injection. For example, soleus electromyographical (EMG) activity appears to recover more rapidly after hindlimb unloading than BTX injection [39], [40]. Further, EMG activity in tibialis anterior increased during hindlimb unloading [39], whereas considerable atrophy was noted after BTX injection compared with other means of achieving disuse [27]. We previously found that weight-bearing ability in the BTX-injected limb was not eliminated and began to recover 2–3 week post-injection [29]. Thus, because of the difference in daily mechanical stimulus in hindlimb-unloaded and BTX-injected limbs, the combined results of these two studies suggest that the role of muscle activity in transmitting vibration is unclear.

Another possible explanation for the absence of a vibration effect is that the vibration stimulus intensity (i.e., the combination of duration, acceleration magnitude, and frequency) may have been insufficient to replace the absence of muscle activity. We only tested a single vibration protocol that we proposed was an approximation of strain signals induced by postural muscle activity, and did not characterize the strains induced by endogenous postural muscle activity. It is possible that varying one or more parameters would have produced a different result. We measured slightly larger peak-to-peak strain magnitudes (5.8 με vs. 2.2 με) for the same frequency and peak acceleration as reported by Garman et al. [21], [22]. However, bone formation responses to low-magnitude stimuli do not appear to be strain-regulated [11], and increasing acceleration magnitudes did not correspond to increased bone formation [41]. Thus, it is unlikely that the subtle difference in strain magnitude had a large effect on our results. Further, postural muscle activity is normally active throughout the day, and we attempted to replace it with only 20 minutes/day. Because we applied the vibration while the mouse was under anaesthesia, it was not possible to apply the stimulus throughout the whole day. However, vibration applied for a long duration (12 hr/d) has been previously shown to negatively alter bone mineralization patterns [42].

There are other factors, aside from strain magnitude, that may be important factors to replace muscle activity with vibration. High-frequency oscillations and/or electrical activity associated with muscle activity are thought to maintain bone homeostasis [3], [17]. However, the highest frequencies for soft tissue oscillation occur in the direction normal to the skin surface [43]. In contrast, we applied the stimulus along the longitudinal axis of the bone. Further, muscle oscillation frequency, recorded with accelerometers [43] and vibromyograms [44], as well as motor unit firing frequency [45] increase with muscle force production. During daily activities, peak force production by postural muscles is relatively constant regardless of the task (e.g., slow walking or fast running) [46]. In contrast, higher force production occurs in muscles with a high percentage of fast-twitch muscle fibres, for more demanding activities such as fast running or jumping. We would, therefore, expect postural muscle oscillations to occur with lower forces with frequency content at the lower end of the frequency spectrum, and that high-frequency oscillations would occur less often. Thus, to accurately mimic postural muscle activity in future studies, stimuli should be applied normal to bone's longitudinal axis at a lower frequency (e.g., 15 Hz).

In addition to the inability to replace muscle activity with vibration, BTX may also have induced an overwhelming osteoclastic response that could not be overcome by the small magnitude vibrations. Most animal studies that observed a positive response to vibration detected small, but significant differences in dynamic histomorphometry, indicating anabolic effects of vibration [7], [8], [9], [21], [22]. In contrast, the results supporting an anti-catabolic effect of vibration are mixed. In separate studies by Xie et al. a similar vibration protocol was noted to have a positive effect [8] and a lack of effect on osteoclastic activity [9]. Further, gene expression patterns in response to vibration are complex as genes associated with both bone formation and resorption were significantly up-regulated [47]. Thus, it is possible that vibration and BTX influence bone remodelling through different, but non-antagonistic signalling pathways.

There is evidence to suggest that both BTX and vibration influence bone through the RANK ligand pathway. RANK ligand (receptor activator of nuclear factor κβ ligand, RANKL) is an osteoclastic differentiation factor that binds to its receptor RANK on the surface of osteoclasts and osteoclast precursor cells [48]. Binding of RANKL to RANK triggers osteoclast formation, fusion, activation, and survival [49]. BTX-induced bone loss likely results from RANKL-mediated osteoclastic activation, as a RANKL inhibitor was able to prevent BTX-induced bone loss [50]. High-frequency, low-magnitude vibration has been shown to suppress soluble RANKL release and RANKL mRNA expression in osteocyte-like cells [51]. However, the upstream regulators of RANKL release could be different in BTX and vibration, or the delay (4 days) between BTX injection and initiation of the vibration protocol may have been too long to prevent RANKL-mediated osteoclastic activation.

To determine whether our vibration parameters were anabolic, we also evaluated the effects of vibration in normal, healthy BALB mice. We did not observe any osteogenic effects of vibration. One of the few studies to demonstrate an anabolic effect of vibration in healthy, skeletally mature mice used the C57BL/6 mice which are known to be more sensitive to mechanical loading [10], [52]. In contrast, Christiansen et al. [14] found that vibration applied without weight-bearing to C57BL/6 mice at 70 or 140 Hz, 0.5 g for 15 minutes/day for 5 weeks was at best weakly anabolic for trabecular bone. Further, in general, BALB mice tend to be less responsive to mechanical stimuli [10] and thus it is possible that the strain of mice used in this study were simply not responsive when healthy. The contrasting results from various studies highlight that vibration effects appear to be species-, protocol- and site-specific [10], [11], [14], [15], [21]. For example, when high-frequency, low-magnitude has been applied to cohorts of post-menopausal women, studies have found increased hip aBMD [53], increased hip aBMD in women who complied with the vibration prescription only [13], and no effect [54]. When combined with resistance training, studies have shown no additional effect of vibration on lumbar or hip aBMD [55], [56]. The variability in results, as well as the lack of understanding of the mechanism underpinning bones' response to vibration makes it difficult to design appropriate interventions to prevent bone loss in post-menopausal women.

The absence of an effect of low-magnitude vibration on muscle mass and cross-sectional area was expected as muscle stimulation is typically used in clinical applications to maximize the paralyzing effect of BTX on normal [57] and spastic muscle [58], [59]. Further, enhanced muscle strength or power has typically been reported following stimulation with high-intensity vibration, greater than 1 g acceleration [53], [60], [61].

In addition to the limitations already described, our conclusions are based on several assumptions. To begin, we assumed that any bone loss that occurred following BTX injection was a result of BTX-induced muscle atrophy. We believe this was a valid assumption as we previously showed that BTX-induced bone loss could be explained by declines in muscle cross-sectional area and that any effect of BTX on bone, independent of its effect on muscle, were likely small or negligible [27]. In addition, mice injected with BTX experienced significant loss of body mass, but the relative loss was similar to what we reported previously [25], despite the addition of daily anaesthesia in the current study. Further, we may have found enhanced bone formation with a longer study duration [8], [9]. However, based on our previous findings, we anticipated the recovery of weight-bearing to begin 2–3 weeks post-injection in BTX-injected animals, and recovery of MCSA to begin 4–6 weeks post-injection. Thus, lengthier application of the stimulus without additional BTX injection would have hindered our ability to apply vibration in the absence of muscle activity.

In conclusion, our data do not support the hypothesis that high-frequency, low-magnitude vibration promotes bone adaptation by mimicking postural muscle activity. In terms of the molecular and cellular mechanisms driving bone formation, recent studies suggested that acceleration of the osteocyte or osteoblast nucleus, rather than matrix strains [21], [62], may trigger the release of osteogenic factors. Further, vibration may promote an osteogenic response by stimulating proliferation and differentiation of mesenchymal stem cells [63], [64], [65]. However, the nature of the endogenous mechanical signal that stimulates these signalling pathways is not yet understood. Given the discrepancy in finding positive effects of vibration between various studies, continued investigation of this mechanism is required to better understand which parameters should be chosen to maximize the therapeutic effect of vibration in humans.
